# Individualized and Clinically Derived Stimuli Activate Limbic Structures in Depression: An fMRI Study

**DOI:** 10.1371/journal.pone.0015712

**Published:** 2011-01-25

**Authors:** Henrik Kessler, Svenja Taubner, Anna Buchheim, Thomas F. Münte, Michael Stasch, Horst Kächele, Gerhard Roth, Armin Heinecke, Peter Erhard, Manfred Cierpka, Daniel Wiswede

**Affiliations:** 1 Hanse Institute for Advanced Study, Delmenhorst, Germany; 2 Department of Psychosomatic Medicine and Psychotherapy, University of Ulm, Ulm, Germany; 3 Institute for Social Therapy, Supervision and Coaching, University of Kassel, Kassel, Germany; 4 Institute of Psychology, University of Innsbruck, Innsbruck, Austria; 5 Department of Neuropsychology, University of Magdeburg, Magdeburg, Germany; 6 Institute of Psychosomatic Cooperation Research and Family Therapy, University of Heidelberg, Heidelberg, Germany; 7 Brain Research Institute, University of Bremen, Bremen, Germany; 8 Brain Innovation, Maastricht, The Netherlands; 9 Department of Psychiatry, University of Bonn, Bonn, Germany; 10 Department of General Psychology II, University of Jena, Jena, Germany; Royal Holloway, University of London, United Kingdom

## Abstract

**Objectives:**

In the search for neurobiological correlates of depression, a major finding is hyperactivity in limbic-paralimbic regions. However, results so far have been inconsistent, and the stimuli used are often unspecific to depression. This study explored hemodynamic responses of the brain in patients with depression while processing individualized and clinically derived stimuli.

**Methods:**

Eighteen unmedicated patients with recurrent major depressive disorder and 17 never-depressed control subjects took part in standardized clinical interviews from which individualized formulations of core interpersonal dysfunction were derived. In the patient group such formulations reflected core themes relating to the onset and maintenance of depression. In controls, formulations reflected a major source of distress. This material was thereafter presented to subjects during functional magnetic resonance imaging (fMRI) assessment.

**Results:**

Increased hemodynamic responses in the anterior cingulate cortex, medial frontal gyrus, fusiform gyrus and occipital lobe were observed in both patients and controls when viewing individualized stimuli. Relative to control subjects, patients with depression showed increased hemodynamic responses in limbic-paralimbic and subcortical regions (e.g. amygdala and basal ganglia) but no signal decrease in prefrontal regions.

**Conclusions:**

This study provides the first evidence that individualized stimuli derived from standardized clinical interviewing can lead to hemodynamic responses in regions associated with self-referential and emotional processing in both groups and limbic-paralimbic and subcortical structures in individuals with depression. Although the regions with increased responses in patients have been previously reported, this study enhances the ecological value of fMRI findings by applying stimuli that are of personal relevance to each individual's depression.

## Introduction

The past decade has seen substantial progress in the search for neurobiological correlates of depression. One major, but not unequivocal, finding is hyperactivity in limbic-paralimbic regions when inducing negative affect, a finding forming part of the cortico-limbic dysregulation model proposed by Helen Mayberg [1]. Limbic hyperactivity might underlie abnormal emotional processing [2] and has been reported in the medial and inferior frontal cortex and basal ganglia (caudate or putamen) during induction of negative affect [3]. An important limbic structure associated with hyperactivity in depression is the amygdala [4], [5]. Studies showing amygdala hyper-responsivity to emotional stimuli have typically used faces [6] or emotional pictures [7]. This activation is thought to be part of an automatic and sustained brain response to negative stimuli, possibly reflecting a bias for negative events in depression [8]. Nevertheless, some studies report no specific amygdala activity in patients with depression when exposed to negative stimuli [9], [10], and findings in the amygdala are reportedly variable [11]. One possible reason for this inconsistency could lie in the nature of the stimulus material utilised. Although often used in basic emotion research, emotional faces or pictures are relatively unspecific and uniform and bear limited relation to clinical features of depression. Further, individual differences in the personal relevance of such stimuli are not taken into account. Importantly, studies using specific and personally relevant emotional words found clear amygdala activation in subjects with depression [8], [12]. Whereas these studies represent an important step forward in the clarification of the role of the amygdala in depression and increase the ecological validity of neuroimaging findings, the use of single words of personal relevance is not a clinically derived means of relating individual psychopathology to brain function.

In order to further enhance the ecological validity of neuroimaging studies of depression, the aim of the current investigation was to expose patients with depression to individually tailored stimuli that specifically activate one significant component leading to or maintaining their depression [13]. Most depressive patients have dysfunctional interpersonal relations as a main feature of their disorder. A clinically reliable measure of interpersonal relations can be obtained via an interview conducted according to the system of Operationalized Psychodynamic Diagnosis (OPD) [14]. Among other valuable clinical information not relevant to this study, an OPD interview yields material pertaining to repetitive dysfunctional interpersonal relations that are specifically involved in the patient's depression. Therefore, it was planned that sentences derived from an OPD interview would be presented to patients and control participants in the fMRI scanner as a means to capture each participant's dysfunctional interpersonal relating. Among other factors, the style of relating reflected in the stimuli is hypothesized to have led to, or to be maintaining the depression in the patients. In controls, the stimuli were designed to consist of sentences reflecting a major interpersonal source of distress. The aim was, hence, to expose participants to their core interpersonal problems while measuring hemodynamic responses of the brain.

Specifically, the following hypotheses were put forward:

- When confronted with their specific dysfunctional interpersonal relations as opposed to unspecific negative stimuli, patients as well as control participants will show increased responses in areas related to emotional processing, conflict monitoring, and self-referential processing (mostly cortical structures of the midline).
- A relative increase of responses in limbic-paralimbic (e.g. amygdala) and subcortical regions (e.g. basal ganglia) is expected when patients are confronted with their interpersonal problems as opposed to the control task and the responses of healthy controls.

## Methods

### Ethics Statement

All participants gave written informed consent after complete description of the study and prior to their inclusion. The study protocol was approved by the ethical committee of the University of Ulm and was conducted according to the principles expressed in the Declaration of Helsinki.

### Participants

Eighteen unmedicated patients with recurrent major depressive disorder and seventeen healthy control participants took part in the study (demographics in Table 1). Patients were recruited from the outpatient department of a psychotherapeutic institute and diagnosed by two trained clinicians (ST and HeK) using the Structured Clinical Interviews I and II for DMS-IV Diagnosis (German version; [15]). Patients reported between 1 and 15 depressive episodes (M[SD] = 5.6[5.5]) and their age at first occurrence of depression was between 8 and 40 years (M[SD] = 19.3[8.2]). Patients had received various types of medication and psychotherapy prior to consulting the aforementioned institute. However, none of the patients had received treatment within at least 6 months prior to inclusion in the study. Exclusion criteria were other psychiatric conditions as main diagnosis, substance abuse, significant medical or neurological conditions (including medical causes of depression), psychotropic medication, and eye problems. Control participants were recruited from the community, matched for age, sex and education and had no history of previous depressive episodes or other psychiatric conditions (SCID). All participants were right-handed. In both groups, depression severity and general symptoms of psychopathology were assessed using the Beck Depression Inventory (BDI, [16]) and the revised Symptom Check List (SCL-90-R, [17]), respectively.

**Table 1:** Participant demographics and behavioral data.

Measure				Controls	Patients	Sign. difference
**Demographics**						
N	total			17	18	
Gender	women: men			14: 3	14: 4	
Age	Mean (SD)			38 yrs (11.6)	39.8 yrs (12.8)	t (33) = .67; n.s.
	Range			22–64	20–64	
Education	Secondary school level I			4	7	
	Secondary school diploma			11	7	
	University			2	4	
**Diagnostics**						
BDI^1^			Mean (SD)	2.2 (2.5)	24.8 (9.3)	t = 9.68; p<.001
			Range	0–9	10–40	
SCL-90-R^2^, GSI^3^,			Mean (SD)	.2 (.1)	1.4 (.6)	t = 6.52; p<.001
			Range	0–.4	.2–2.5	
Post Scan rating		sentence adequacy	Mean (SD)	5.9 (.7)	5.8 (.9)	t = .20; n.s.
			Range	5–7	4–7	
		sentence arousal	Mean (SD)	4.8 (.7)	5.1 (1.0)	t = 1.16; n.s.
			Range	4–7	3–7	
PANAS^4^		Pre Scan Positive Affect	Mean	30.0 (5.7)	25.9 (6.5)	See text.
			Range	18–39	14–37	
		Post Scan Positive Affect	Mean	27.9 (7.2)	25.5 (7.7)	
			Range	14–41	12–37	
		Pre Scan Negative Affect	Mean	11.7 (1.5)	16.8 (4.4)	
			Range	10–15	10–29	
		Post Scan Negative Affect	Mean	10.7 (1.3)	15.3 (6.1)	
			Range	10–15	10–29	

Abbreviations: 1: BDI = Beck Depression Inventory, 2: SCL-90-R = Symptom Check List Revised, 3: GSI = Global Severity Index, 4: PANAS = Positive and Negative Affect Schedule.

### Stimuli

To assemble individualized and personally relevant stimuli that related to depressive symptoms, an OPD interview (Operationalized Psychodynamic Diagnosis) [14] was conducted with each patient and each control participant. OPD is a multiaxial system assessing psychopathology on several levels [18]. Beyond a pure description of symptoms (Axis V), it includes experience of illness (Axis I), dysfunctional interpersonal relations (Axis II), psychodynamic conflicts (Axis III) and psychological structure (Axis IV) [14]. Although OPD is at its core a psychodynamic approach, dysfunctional relations (Axis II) are considered by most therapeutic schools to be important in the development and maintenance of depression [19], [20]. The OPD interviews were conducted by a trained clinician (HeK) and videotaped. Dysfunctional relations were rated independently by 2–3 expert raters blind to the status of the interviewees. Although not suffering from depression, control participants also experienced dysfunctional relations which reflected a major interpersonal source of distress. From the systematic and item-based diagnosis [14] four sentences were identified representing the core dysfunctional relationship theme of each person (e.g. “You wish to be accepted by others.”, “Therefore you do a lot for them.”, “That is often too close for them, so they retreat.”, “Then you feel empty and lonely.”). It is important to notice that these sentences do not represent “depression” or “negative mood” in general, but intentionally point to a significant and specific aspect of each individual's depression – its development and/or maintenance. These individual sentences served as stimuli during the fMRI-session (OPD condition). Word count and semantic structure of the stimulus sentences (i.e. distribution of the thirty-two items assigned) did not differ between patients and controls.

The control condition was termed “traffic” and comprised four sentences describing stressful traffic situations. Participants were instructed to recall a stressful traffic situation they had experienced whilst reading the “traffic” sentences. The rationale behind this control condition was to induce negative emotions and recall autobiographical memories with a personally relevant situation including human interactions, but without engaging in material that might interfere with participants' depression or interpersonal distress.

In order to separate the two conditions (OPD and traffic), and allow subjects the opportunity to recover after emotionally demanding sentences, “relaxation” sentences were inserted between conditions. These sentences instructed participants to relax by thinking of a safe place. Subjects were prepared for the “relaxation” condition before the experiment.

Whereas the OPD sentences were derived individually for each person, “relaxation” and “traffic” were the same sentences across all subjects. OPD sentences were slightly but significantly longer (M[SD] = 49.8[9.1] characters) than “traffic” sentences (43.5 characters, p<.001). There was, however, no significant difference in length between the OPD sentences for patients and controls. All sentences were presented in German.

### fMRI Tasks

The four sentences of each condition (OPD, traffic, relaxation) were individually presented for 7.5 seconds while subjects were in the scanner. During the OPD block participants were asked to mentally engage in situations with significant others, as described by the OPD sentences. Subjects received no instruction to regulate their emotions, but were instructed to allow spontaneous thoughts, emotions and memories come to mind. According to the logic of the OPD Axis II, the four sentences comprising the dysfunctional interpersonal relation form one complex that should activate a specific and disorder-related mental representation [13]. Therefore, the four sentences were modelled as a whole in fMRI analyses. “Traffic” and “relaxation” conditions also comprised four sentences each lasting 7.5 seconds. The instructions were to mentally engage either in the recalled traffic situation or to relax. In total, 12 “relaxation”, 6 “traffic” and 6 “OPD” blocks were presented. Blocks were separated by a 5-seconds fixation cross. The entire experiment lasted approximately 15 minutes.

### Procedure

Four to six weeks prior to fMRI assessment, participants were interviewed (SCID I+II, OPD), completed questionnaires (BDI, SCL-90-R) and gave written consent to participation. At the beginning of the fMRI session and prior to scanning, subjects were presented with their individual OPD sentences and asked whether the sentences adequately represented their problematic relations. To control for state affectivity, all participants filled out the German version of the Positive and Negative Affect Schedule (PANAS; [21], [22]) before entering the scanner. After scanning, a second PANAS was completed together with a questionnaire assessing on a 7-point Likert scale the extent to which the OPD sentences were correct and caused emotional arousal.

### Image Acquisition

MRI data were recorded (DW and PE) using a 3-T SIEMENS Magnetom Allegra head scanner (Siemens, Erlangen, Germany). Subjects were positioned on the scanner couch and wore foam earplugs to reduce scanner noise. An experienced psychotherapist (ST or HeK) assisted with the setup procedure and spoke to the patients both prior to and after the experiment. Further, the therapist explicitly asked the subjects whether they were fully awake and ready to continue in the break between the scanning sessions. Data acquisition started with anatomical images (3D high resolution T1-weighted isotropic volume, MPRAGE-sequence (MPRAGE = Magnetization Prepared Rapid Gradient Echo; [23]); TR = 2.3 s, FOV = 256×256×176 mm, TE = 4.38 ms, TI = 900 ms, flip angle = 8°, 1 mm isovoxel, total acquisition time 14.45 min). Functional scans were performed using a single shot echo planar imaging sequence (EPI). A total of 365 T2*-weighted whole brain volumes were acquired (EPI-sequence; TR 2500 ms, TE 30 ms, flip angle 90°, FOV 192 mm, matrix 64×64, 44 slices, slice thickness 3 mm, interleaved acquisition order, AC-PC- Orientation, total acquisition time: 15.18 min).

### fMRI data analysis

Data were analyzed and visualized using Brain Voyager QX 1.10 (Brain Innovation, Maastricht, Netherlands). Preprocessing: Functional data were slice-time corrected, motion parameters were estimated, and motion was corrected relative to the first volume of the run. To remove low frequency drifts, data were high-pass filtered (3 cycles, three sine waves fall within the extent of the data). Structural and functional data were transformed into the standard space of Talairach and Tournoux [24], data points were labeled using Talairach Daemon [25]. The design matrix was modeled using the two gamma hemodynamic response function. Functional data were smoothed using an 8 mm full width at half maximum (FWHM) isotropic Gaussian Kernel.

Statistics: Group data were analyzed using random effects analyses based on z-transformed functional data. An ANOVA, including the within-factor CONDITION (OPD vs. traffic sentences) and between-factor GROUP (patient vs. control) was performed to identify differences in hemodynamic response. Separate brain maps were generated for the main effect CONDITION and GROUP and for the interaction CONDITION x GROUP. The main effect of CONDITON is displayed as a t-statistic, which yields the same results as the F-statistic, but allows color-coding the direction of changes. Motion-correction parameters were included in the GLM-Model. Maps are shown with a threshold of p<0.001. Correction for multiple comparisons for the within-factor CONDITION was based on False Discovery Rate (FDR) [26]. However, literature suggests differences between controls and patients in relatively small cortical and subcortical regions [3], but FDR is very strict for small active areas. Thus, the between-factor GROUP and the interaction are reported on p<.001 (uncorrected). For all reported comparisons, the likelihood of Type I error was reduced based on cluster size threshold estimation [27], [28] involving a Monte Carlo simulation calculating the likelihood to obtain different cluster sizes. Calculations resulted in a cluster size threshold of 16 voxels. Active voxels are displayed in native resolution without interpolation and plotted on the Talairach-transformed brain.

## Results

### Behavioral Data

**Figure 1:**
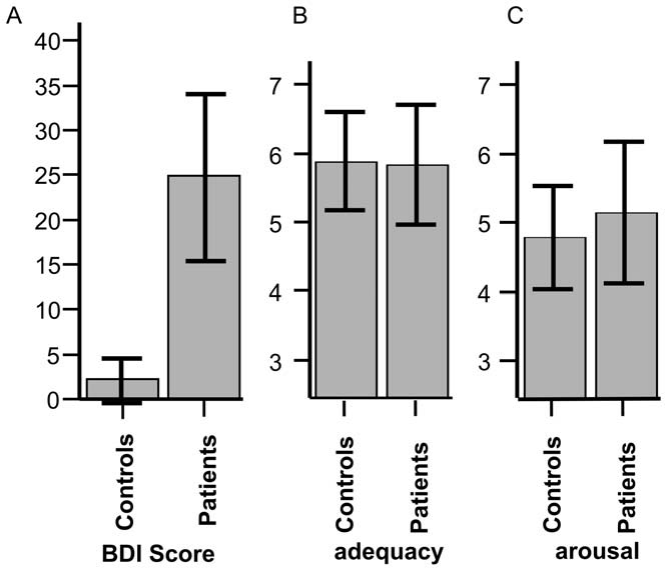
Depression and impact of OPD sentences. A: BDI (Beck Depression Inventory) scores for all subjects and group data. B and C: Scales display whether the OPD sentences were adequate for the participant (B) and whether participants where emotionally aroused by the OPD sentences (C). Error bars show +/− 1 SE.

Table 1 shows behavioral data for patients and controls. Patients had significantly higher depression scores (BDI, Table 1; Figure 1) and general symptoms of psychopathology (GSI-scale of the SCL 90-R). Both groups judged the OPD sentences to be adequate descriptions of their dysfunctional interpersonal relations. After the fMRI session, all participants reported that the OPD sentences caused emotional arousal in the scanner. There were no significant differences between groups in terms of adequacy or arousal induced by the OPD sentences (Table 1; Figure 1). Patients with depression had significantly higher levels of negative affect as measured by the PANAS, and there was a tendency toward reduced negative affect after completion of the fMRI session. This tendency was seen in both groups (main effect GROUP: F(1,29) = 16.38; p <.001; main effect Pre/Post fMRI; F(1,29) = 3.35; p<.077, interaction n.s.). There were no differences between groups in positive affect as measured by the PANAS prior and after the fMRI session (PANAS positive affect, main effect GROUP, Pre/Post fMRI and interaction n.s.). See Table 1 for mean values. PANAS ratings in both groups were comparable to normative data obtained from a large group of healthy subjects under stress-free conditions [21].

### Neuroimaging Results

**Figure 2:**
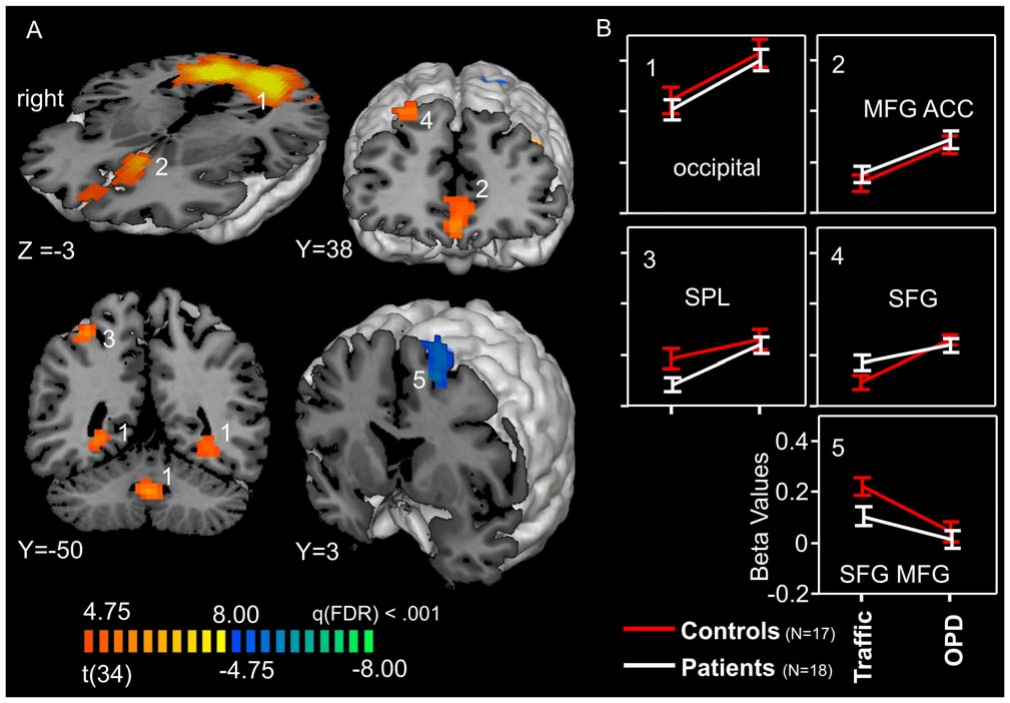
Main effect of *CONDITION*. A: t-maps, p<.001, FDR, cluster threshold 16 voxels. B: Beta plots for regions with significant main effect, orange-yellow-scale: OPD>Traffic, Blue Scale: Traffic>OPD. Error bars show +/− 1 SE; coordinates are provided in Talairach space, abbreviations as in Table 2.

The main effect CONDITION, displayed as a t-contrast, identified regions with a stronger signal for OPD relative to traffic sentences. These regions were located in the occipital cortex, in the superior parietal lobe, the superior frontal gyrus, in the anterior cingulate cortex (ACC), and in the medial frontal gyrus. Conversely, a stronger signal for Traffic relative to OPD sentences was observed in a cluster including parts of the superior and middle frontal gyrus (Table 2; Figure 2).

**Table 2:** Areas significant for the Main Effect CONDITION and interaction CONDITION x GROUP.

Main Region	Cluster size	X	Y	Z	t or F	Side	Regions	BA^1^
**OPD>Traffic**								
occipital	79785	−4	−73	0	10.48	R	Cerebellum	
						R	Cuneus	18,17,30,23,7
						R	Lingual G.^2^	18,19,17
						R	Posterior Cingulate	30,31,23
						R	Precuneus	31,23
						R	Fusiform G.	19,37
						R	Middle Occipital G.	18,19
						R	Parahippocampal G.	19,30
						L	Cerebellum	
						L	Lingual G.	18,19,17
						L	Middle Occipital G.	18
						L	Cuneus	18,17,30,23,19
						L	Fusiform G.	19,18,37
						L	Posterior Cingulate	30,31,23
						L	Inferior Occipital G.	18,19,17
						L	Precuneus	31,23
						L	Parahippocampal G.	19,30,37,18
SPL^3^	621	31	−48	52	6.05	R	Superior Parietal Lobule	7
						R	Precuneus	7
						R	Inferior Parietal Lobule	40
SFG^4^	540	21	38	48	5.72	R	Superior Frontal G.	8
MFG^5^/ACC^6^	4752	−2	40	1	6.78	R	Medial Frontal G.	10
						L	Anterior Cingulate	32,24,10
						L	Anterior Cingulate	32,24
						L	Medial Frontal G.	10,11
**Traffic>OPD**								
SFG^7^/MiFG^8^	2133	−21	3	58	−6.46	L	Superior Frontal G.	6
						L	Middle Frontal G.	6
						L	Medial Frontal G.	6
**CONDITION x GROUP Interaction**								
IFG^9^	702	51	14	2	15.25*	R	Inferior Frontal G.	45,47
						R	Precentral G.	44
						R	Superior Temporal G.	22
Amygdala	432	23	−3	−17	21.86*	R	Amygdala	
MFG	540	21	−3	51	18.94*	R	Medial Frontal G.	6
R. Putamen	2592	16	18	−7	27.2*	R	Putamen	
						R	Caudate Head	
						R	Lateral Globus Pallidus	
						R	Inferior Frontal G.	47
						R	Middle Frontal G.	11
L. Putamen	2538	−15	17	−8	21.21*	L	Putamen	
						L	Caudate Head	
						L	Subcallosal G.	34,47
						L	Inferior Frontal G.	47
Prec.G./MiFG	837	−33	−7	47	20.01*	L	Precentral G.	6
						L	Middle Frontal G.	6
Postc.G.	432	−40	−29	44	17.24*	L	Postcentral G.	2,40
						L	Inferior Parietal Lobule	40

Legend: Areas which are significant for the Main Effect CONDITION are reported at the level of p<.001 (FDR, cluster-threshold 16 voxels) and areas for the interaction CONDITION x GROUP at the level of p<.001 (cluster-threshold 16 voxels). X,Y, Z values indicate center of gravity of the cluster in Talairach-space. Number of voxels gives the number of active voxels in this specific region and/or in this Brodmann area. Column “t or F” represents maximal t-value or F-Value (indicated by *) for the given cluster. See also Figures 2 and 3.

Abbreviations: 1: BA = Areas according to Brodmann, 2: G. = Gyrus, 3: SPL = Superior Parietal Lobule, 4: Superior Frontal Gyrus, 5: MFG = Medial Frontal Gyrus, 6: ACC = Anterior Cingulate Cortex, 7: SFG = Superior Frontal Gyrus, 8: MiFG = Middle Frontal Gyrus, 9: IFG = Inferior Frontal Gyrus.

**Figure 3:**
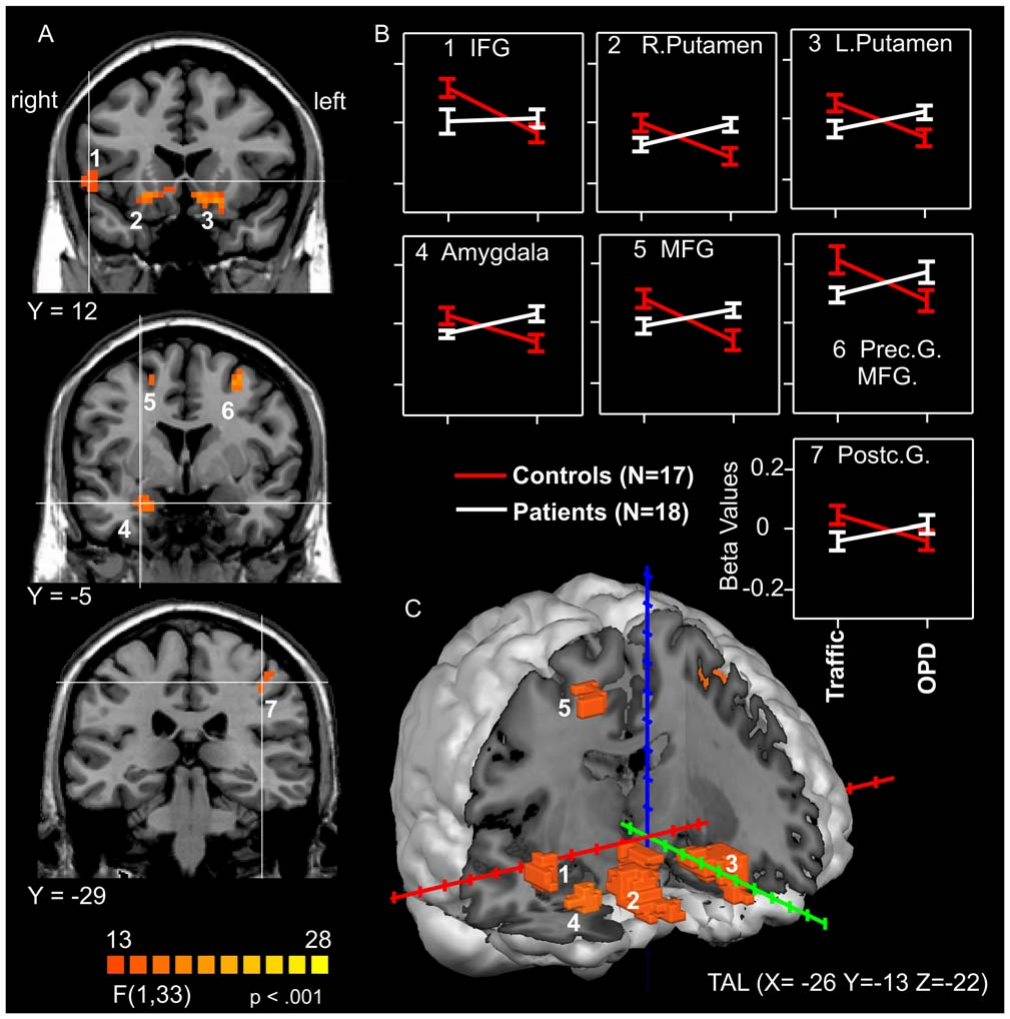
Interaction effect *CONDITION* x GROUP. A: Brain slices depict coronar view of the active clusters, p<.001, cluster threshold 16 voxels. B: Interaction plots for active clusters, based on beta values for OPD and traffic sentences. C: all active areas are projected into the brain. Error bars show +/− 1 SE; coordinates are provided in Talairach space, abbreviations as in Table 2.

A significant group by condition interaction was found in a variety of regions, including the inferior frontal gyrus, the postcentral gyrus, the amygdala, the precentral and middle frontal gyrus, and the basal ganglia. In general, the hemodynamic response pattern can be described as a signal increase for patients when confronted with OPD relative to Traffic sentences. In contrast, controls show a signal decrease for this comparison (Table 2; Figure 3).

Central conclusions are derived from the GROUP X CONDITION interaction reported above. In order to establish whether the traffic condition itself might yield differences between groups, a “TRAFFIC minus RELAX” comparison was performed between groups. This comparison addressed the potential confound that would be introduced should patients with depression be shown to exhibit stronger responses in limbic areas in the traffic condition - potentially due to a negative bias in handling emotional stimuli in this condition. However, the comparison revealed no active clusters, showing no differences between patients and controls in the traffic situation. In a similar vein, groups were compared based on the difference “OPD minus RELAX”. OPD relative to RELAX sentences led to stronger BOLD responses in seven clusters, including the amygdala, the inferior/middle frontal gyrus, the right and left postcentral gyrus, showing a large overlap with areas that have been reported for the interaction GROUP X CONDITION.

## Discussion

This study compared hemodynamic responses in the brains of patients with depression to those of matched healthy control participants. The experiment utilised individually tailored, yet highly standardized stimuli thus enhancing the ecological validity of fMRI findings. Although individualized stimuli have been used in neuroimaging studies with PTSD patients [29], a control condition with emotionally arousing, personally relevant but not disease-specific content (“traffic”) has not been included in comparable experimental designs so far.

Across both groups, the presentation of individualized sentences describing dysfunctional interpersonal relations led to increased hemodynamic activity in the ACC, medial frontal gyrus, fusiform gyrus and large portions of the occipital lobe. Thus, the newly described paradigm yielded plausible responses in areas related to emotional processing, perspective-taking, mentalizing and self-referential processes, confirming our first hypothesis. When confronted with the interpersonal stimuli, patients with depression, when compared to healthy controls, displayed increased hemodynamic activity in limbic-paralimbic and subcortical structures including the amygdala. This confirmed our second hypothesis and lends further support to the model of limbic hyperactivity in depression by the use of ecologically valid stimuli.

Bilateral regions of the ACC and medial frontal gyrus, which form part of the cortical midline structures, showed enhanced hemodynamic responses during the personally relevant OPD condition. Cortical midline structures have often been associated with the processing of self-referential stimuli [30]. Hence, consistent responses of these areas point to the self-relevance of the OPD condition. Interestingly, another study has reported activation in the ventral part of the medial frontal gyrus in patients with depression and controls when judging self-relevant attributes [31]. The involvement of the ACC and medial frontal gyrus in emotional processing is well established [32] with the ACC hypothesized to play a key role when attending to subjective emotional responses [33]. Importantly, the area of ACC activation in the current study lies in the affective division [34] and might therefore reflect the inherently higher emotional load of the OPD condition as opposed to the traffic condition.

There are several explanations for the more consistent hemodynamic responses in bilateral visual cortex in the OPD condition. Firstly, the OPD sentences are of enhanced personal relevance and, therefore, should have enhanced potential to trigger vivid mental images. Increased mental images are also thought to underlie the greater activity of visual areas in response to concrete relative to abstract words [35]. Furthermore, in a meta-analysis of studies analyzing emotional processing almost half of the studies comparing emotional with neutral conditions showed enhanced activity in visual cortex [32]. This is believed to reflect emotional arousal acting upon visual areas to enhance perception of salient stimuli [32]. For instance, the fusiform gyrus, an area hemodynamically active in our study, shows enhanced responses upon presentation of visual stimuli (faces) depicting danger [36]. Although both conditions, traffic and OPD, can be regarded as emotional, the salience and emotional load of OPD sentences should be inherently higher since they are derived from each participant's core problematic relation.

It is of note that amygdala responses, which have been obtained only inconsistently with non-individualized emotional stimuli in previous studies [3], [4], [5], [11], were very robust in our task. Two previous studies using stimuli of personal relevance (words) have also found amygdala responses in subjects with depression [8], [12]. In these studies, critical word stimuli were generated by participants who were asked to find words that “best represent what [they] think about when [they] are upset, down, or depressed” [8]. Our stimuli depicting problematic relationships could further increase the ecological validity of neuroimaging findings by activating content that is tied to each individual's experience of depression. We speculate that enhanced hemodynamic amygdala activity in subjects with depression reflects their higher emotional involvement in problematic relationships.

In line with our results in the amygdala, areas of the putamen and caudate nucleus also showed increased hemodynamic responses in patients when engaging in the OPD condition. According to a recent meta-analysis, the basal ganglia have consistently displayed increased hemodynamic activity in depression after induction of negative affect [3]. This is not surprising, given that the basal ganglia have rich interconnections with limbic structures (including the amygdala) and prefrontal areas, and form part of multiple cortico-subcortical loops engaged in reward, punishment, affect and motivation [37]. In line with this, the basal ganglia are increasingly discussed as a target location in the context of deep brain stimulation for the treatment of depression [38].

Among other areas exhibiting selective responses in the patient group were the inferior and middle frontal gyrus and the inferior parietal lobule, findings in line with a recent meta-analysis [3]. However, the exact role of these areas in the psychopathology of depression is largely unknown currently. It is perhaps unsurprising that no differential response was observed between patients and controls in dorsolateral prefrontal areas since prefrontal abnormalities might mainly be responsible for the cognitive deficits in depression [39]. Further, hypoactivity in dorsolateral prefrontal areas has been the least consistent finding in emotional activation studies [40].

### Limitations

Several limitations of this study deserve mention. Firstly, while the use of OPD sentences as the main stimuli is the genuine strength of this study, it is also the major source of potential confounds. It is impossible to know what subjects are actually thinking of when instructed to mentally engage in the problematic interpersonal relation depicted by the OPD sentences. If one follows the logic of the OPD system and interpersonal theories of depression, the stimuli are highly specific and directly related to a significant factor contributing to the development and maintenance of depression. On the other hand, they are less controllable in terms of what reactions they produce in subjects than standardized and widely-used stimuli such as IAPS pictures [41]. A further possible confound lies in the traffic-related sentences as a control condition. It has been reported that patients with depression show a negative bias in the evaluation of emotional stimuli, which could lead them to react to unspecific traffic-related stress with enhanced hemodynamic responses in brain areas involved in the processing of (negative) emotions (e.g. limbic structures). In order to ensure that the limbic responses found in the interaction GROUP X CONDITION were caused by the OPD sentences themselves we conducted additional comparisons demonstrating that the contrast “TRAFFIC minus RELAX” yielded no differences between patients and controls and further, that the contrast “OPD minus RELAX” indicated hemodynamic activity in amygdala and inferior/middle frontal gyrus in the patient group. This suggests that the interaction effect GROUP X CONDITION is not driven by differences between groups in the TRAFFIC condition. Finally, another potential problem may lie in the standardized style of the TRAFFIC sentences. Thus, it could be the case that brain responses reflect the difference between personalized and general stimuli. This limitation lies in the study design and could not be ruled out by additional analyses.

### Conclusion

The present fMRI study describes clear differences in hemodynamic responses between patients with depression and non-depressed control participants using personalized stimuli in a highly standardized fashion, thus supporting the model of limbic hyperactivity in depression. The stronger response in the amygdala and basal ganglia found for OPD sentences in patients could indicate particular involvement of these structures in the processing of clinically derived and personally relevant material. Increased responses in cortical midline structures when confronted with problematic interpersonal sentences suggests that our novel experimental design engaged both groups of participants in self-referential and emotional processing.
